# Fadi Meroueh: responding to the health needs of prisoners

**DOI:** 10.2471/BLT.21.030121

**Published:** 2021-01-01

## Abstract

Fadi Meroueh talks to Andréia Azevedo Soares about ensuring health equity and harm reduction services in prisons, and the challenges presented by the COVID-19 pandemic.

Q: What are the main differences you have encountered working as a doctor outside and inside prisons?

A: For me one of the biggest differences is the way doctors are pressured into different and often conflicting roles inside correctional facilities. This can make it difficult for the doctor to retain independence and loyalty to the patient. For example, magistrates may ask doctors to certify that an inmate can make a court appearance. In my view such requests should be refused. If a judge or a lawyer wants to know whether a patient is prepared to go to court, he should hire an expert in such matters to make that assessment. Similarly, prison guards have a tendency to ask for information about a prisoner’s health as though the doctor were part of the prison’s security staff, something I find completely unacceptable.

Q: Does that often happen?

A: Absolutely. It has happened to me when I have called the guards to say a patient needs to go to the emergency room. They ask me what is wrong with the prisoner – usually because they have security concerns. But should I tell them? No. Prisoners may be deprived of their freedom but not of their health rights. Medical privacy and independence of medicine are key principles, and the struggle to maintain them in prison has been going on for 30 years in France. It is a long battle. Another difference is the way doctors and patients tend to relate to each other inside prisons. When someone is incarcerated, they lose contact with friends and family. Their lives become the penitentiary and they are often enclosed in a cell which may measure just a few square metres. As a result, there is a different doctor–patient dynamic in the consulting room. It is of course the doctor’s job to be concerned with the patient’s well-being, and to ask about any physical or mental concerns they may have. However, the doctor is not there to be the patient’s friend. He or she is the patient’s doctor and should behave as one would in the broader community.

Q: You were elected president of Health Without Barriers in 2019. What is the purpose of that organization?

A: Health Without Barriers was created to bring together and represent professionals dealing with health care in prisons. Our mission is to improve inmates’ health through the promotion of good health practices and ethical standards for the safeguard of human rights in prison. We also encourage and support research and interdisciplinary collaboration in the field of prison health. We have a broad remit covering most health-care interventions from dental to mental, but key areas of concern include drug addiction and the prevention of infectious disease transmission. With regard to the latter, we are currently collaborating with several European and global organizations and are part of two European projects: the European Centre for Disease Prevention and Control project which is focused on the prevention of infectious diseases in penal institutions;**and the Prison and Hepatitis C in Europe****project that is working on developing the evidence base and implementing effective diagnosis and management of chronic hepatitis C among inmates in European prisons.

“Prisoners may be deprived of their freedom but not of their health rights.”

Q: Prisons are often discussed as incubators of infectious disease, including diseases associated with needle use. What is your perspective on that issue?

A: I think it’s important to understand that – while the prevalence of HIV (human immunodeficiency virus), HCV (hepatitis C virus), HBV (hepatitis B virus) and tuberculosis is higher in prison populations than in the general population – in many instances, prisoners have a drug problem before they are incarcerated. Indeed, they are often incarcerated because of problems deriving from their drug use such as drug possession offences, theft, etc. So, inevitably prisons tend to see a higher concentration of needle users.

Q: You started a needle and syringe exchange programme at Villeneuve-lès-Maguelone. Could you tell us about that experience?

A: When I started the needle exchange programme here in 2010, I realized that there would be little point in treating prisoners with HIV, HCV and HBV without providing them with clean syringes. However, despite French laws regarding the implementation of needle exchange programmes in prisons, many doctors wait for a go-ahead from the prison administration to start implementation. We didn’t do that here. We order needles in the same way we order other medical supplies because we take the view that harm reduction is a legitimate evidence-based health intervention. Unfortunately, penitentiary staff are still resistant.

Q: Why is that?

A: For different reasons, but one of them is the fear of staff being attacked with infected needles. The fear is rooted in an incident that happened in Australia in 1990 at the Long Bay Jail in Sydney, when a guard was stabbed with an HIV-infected syringe and subsequently died of an AIDS-related illness. Since then, politicians and other decision-makers have refused to talk about needle and syringe exchange programmes. It really is taboo. The reality is that the risk of needle-stick injury for prison staff will exist as long as needles are in circulation, regardless of whether needle exchange programmes are in place. My argument is always the same: the day needles cease to be used, needle exchange can stop. Resistance to needle exchange is not solely a French phenomenon, of course. Since 1993, when WHO gave its support to this type of harm reduction programme in detention centres, only a handful of countries have implemented them.

Q: You and your team have managed to eliminate Hepatitis C at Villeneuve-lès-Maguelone. What was the key to your success?

A: Having a proactive treatment policy. We used to wait for patients to be released from prison before ensuring that they were treated outside because treatment took from six months to one year and many of our inmates serve terms shorter than that. Since 2003 we have been treating every patient infected whether they are staying in prison for one month or a year. Also, we do not wait for severe symptoms to initiate treatment. By beginning treatment early, we not only improve outcomes, we also improve treatment adherence. People who have made a start in prison tend to continue outside. Of course, the arrival of new antiviral treatments since 2014 has also revolutionized treatment, making it possible to complete the full course in two months.

“People considered vulnerable to severe COVID-19 who hadn’t committed serious crimes were released.”

Q: How has COVID-19 impacted your prison?

A: It has been challenging. We knew we had to take it very seriously because like any other prison we bring together a large group of people in an enclosed space and are thus vulnerable to the transmission of any respiratory disease. Moreover, we were about 50% over-capacity at the beginning of 2020, with around 900 inmates under our roof. We actually had people sleeping on mattresses on the floor! Starting in March 2020, we implemented strict transmission prevention measures. Visits and activities were suspended, and detainees had to spend most of their time in their cells, which was very hard on them psychologically. We also took steps to reduce overcrowding. Detainees with sentences up to 2 months were released earlier, and people considered vulnerable to severe COVID-19 who hadn’t committed serious crimes were released. We managed to get the population down to 729 detainees, which is still above maximum capacity but is a significant improvement. As a result of these efforts, in the first half of 2020 only two inmates tested positive for the virus.

In the second phase of restrictions, which was instituted in November 2020, we were better prepared and were able to be less restrictive of people’s movement and were also able to maintain visits, which is very important from the point of view of prisoners’ mental well-being. The fact that the quarantine period for people who have been exposed to COVID-19 has been reduced to 7 days from 14 has also helped, and we now have rapid antigen tests that in 15 minutes give reasonably reliable results if they are used within 5–7 days of the onset of symptoms. We are still being very strict about masks with everybody being obliged to wear a mask all the time. Also, everyone arriving from the outside has to go into quarantine. So far these measures are working quite well. As of mid-November 2020, only two of the inmates had tested positive in the second half of the year.

Q: What are your main concerns going forward?

A: Making sure we continue to be vigilant with regard to COVID-19 transmission. We cannot let our guard down, regardless of recent developments with vaccines. We also need to make sure that prisons get the resources they need. Because of the impact of response measures on the economy, an economic crisis is around the corner which is going to put tremendous pressure on the government to cut costs. We have to make sure that we do not try to save money at the expense of prisoners' health. First because health is their right. Health is health for all. But even at the more pragmatic level of good health-system governance, it is clear that prisoners’ health should be a concern for everyone. Most prisoners eventually rejoin society and their successful reintegration depends in part on their good health. If former detainees return to society with an infectious disease, they are likely to increase disease transmission and the community’s overall disease burden. And they will still require treatment. So why not make sure they are treated in prison?

There also needs to be more investment in mental health treatment capacity, including in the facilities required to accommodate the people who need them. We have to stop using prisons for patients with psychosis or schizophrenia who have committed minor offences. Another concern with regard to the coming downturn that most economists are predicting is that it is likely to impact the marginalized hardest. Many of the people who come to prison, come in precisely because they are marginalized and suffering from inequities. If we do not try to address socioeconomic marginalization with investment in education and job creation, people will keep being incarcerated.

